# Delayed Symptomatic Entrapment and Herniation of Lumbar Nerve Root Due to a Prior Durotomy Defect Initially Misdiagnosed As Arachnoiditis: A Case Report

**DOI:** 10.7759/cureus.28588

**Published:** 2022-08-30

**Authors:** Caren M Stuebe, Jose M Soto, Awais Z Vance

**Affiliations:** 1 Surgery, Texas A&M School of Medicine, Temple, USA; 2 Neurosurgery, Baylor Scott & White Medical Center, Temple, USA

**Keywords:** herniation, radicular pain, duraplasty, durotomy, case report

## Abstract

Incidental durotomies are well-known complications of spine surgery. They are often identified and repaired intraoperatively, with a preference for primary rather than secondary repair. We present the case of a middle-aged male with worsening radicular pain six months after spinal surgery complicated by a durotomy defect. His pain was worse with coughing or standing. Magnetic resonance imaging identified an L3-L5 extradural fluid collection in the lumbar spinal canal and an empty sac sign. Computed tomography lumbar myelogram identified clumping of the cauda equina nerve roots at L2-L3 and an empty sac sign at L4-L5 and L5-S1, concerning adhesions and arachnoiditis. The patient's unusual worsening of symptoms and a history of a durotomy defect with secondary repair led to suspicion of an alternative cause. Surgical exploration identified the left L5 nerve root herniated through the durotomy defect. Reduction of the nerve root herniation with primary repair of the durotomy was performed, and the patient experienced immediate relief that was stable at his one-month follow-up. This case features an unusual presentation of a delayed herniated nerve root through a prior durotomy defect with entrapment. We highlight the importance of a high degree of caution in cases of increased radicular pain following spinal surgery with a known durotomy, particularly when symptoms do not support the clinical presentation of arachnoiditis. Additionally, primary repair of durotomies should be undertaken whenever possible to avoid this potential complication.

## Introduction

An incidental durotomy is an unintended tear or puncture of the dura mater that frequently occurs during spinal surgery, varying from 2.9% to 14% [1-3]. Incidental durotomies are thought to be more common at spinal levels with scar tissue from prior surgery or irradiation [4, 5]. Labaran et al. reported an incidence of 3.7% in patients with a history of lumbar epidural steroid injections [6]. Treatment is surgical repair, involving either primary closure with sutures and an optional patch or secondary closure with an on-lay dural substitute. Computed tomography (CT)-guided percutaneous patching has also been a suggested treatment, especially in cases requiring a second procedure with a dural defect less than 5 mm and the absence of a pseudomeningocele [7].

Surgical repair of durotomies typically results in good clinical outcomes, with some reporting comparable long-term outcomes between surgical patients with and without durotomies [1, 8-11]. Short-term postoperative sequelae include headaches and wound infection, while cerebrospinal fluid (CSF) leaks, pseudomeningoceles, neurologic deficits, and arachnoiditis are the most common long-term postoperative sequelae [3]. However, there is limited literature on the clinical outcomes of incidental durotomies when stratifying surgical repair by primary versus secondary closure. 

We report a case of a herniated and entrapped nerve root through a prior durotomy defect with an initial secondary repair. A delayed presentation, imaging findings suggestive of arachnoiditis, and confirmation of the diagnosis only upon surgical exploration make this a complex and exciting presentation of a relatively common complication of spinal surgery with uncommon postoperative sequelae. This case report has been in line with the surgical case report (SCARE) criteria and has an Institutional Review Board (IRB) exemption because it has no patient identifiers. Patient consent was not required by our ethics committee [12].

## Case presentation

**Figure 1:**
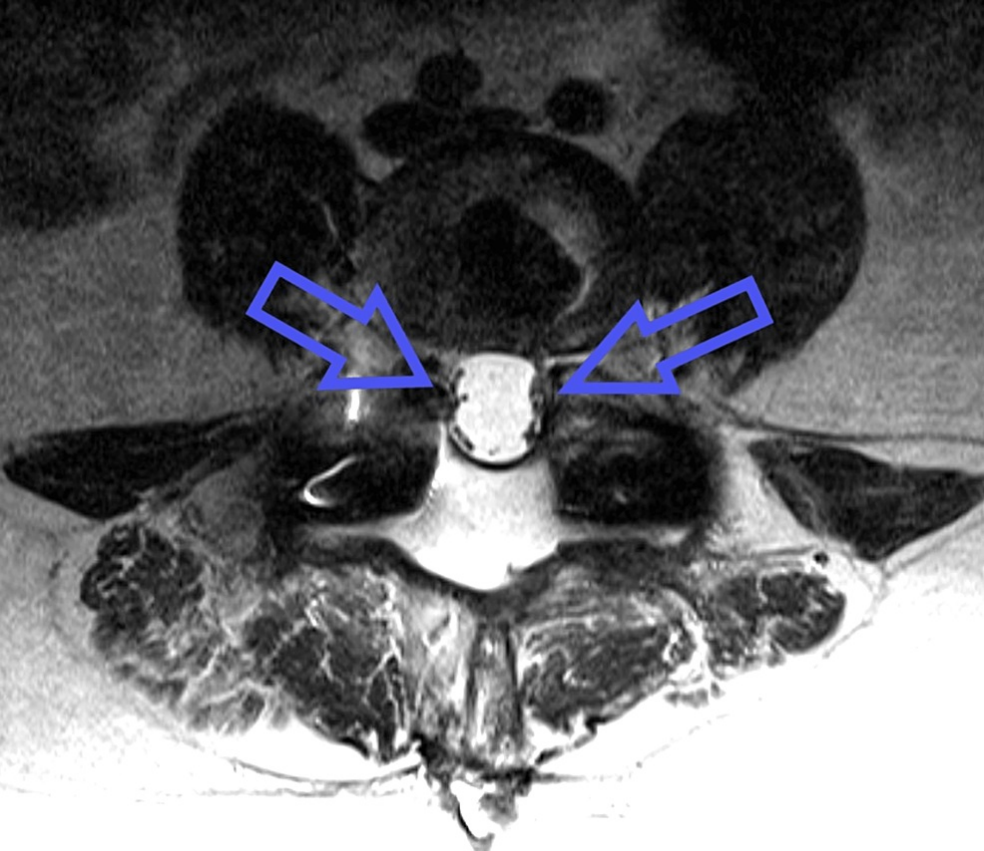
T2-weighted magnetic resonance imaging of the lumbar spine at L4-5 demonstrating clumping of the nerve roots (arrows) to the edges of the dura bilaterally (i.e., an "empty sac sign").

**Figure 2:**
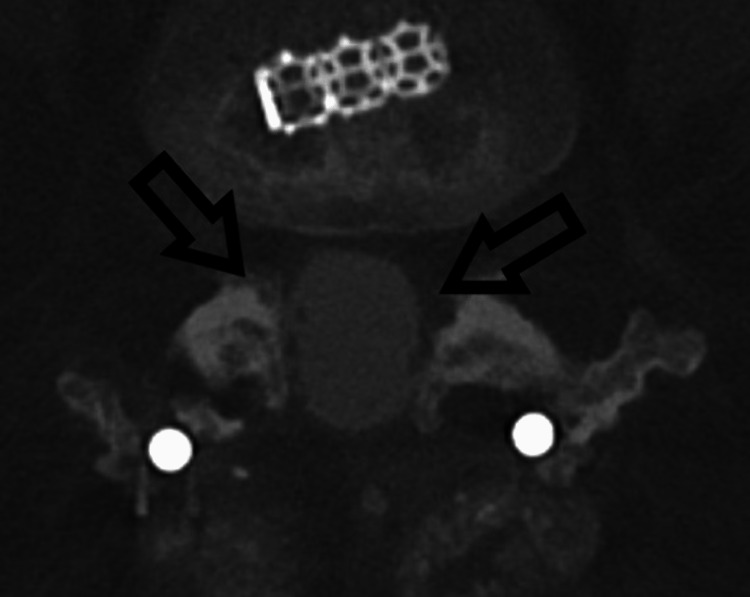
Computed tomography lumbar myelogram also demonstrates apparent adhesion of the lumbar nerve roots to the peripheral aspect of the dura (arrows).

A 45-year-old male with a history of chronic back pain presented to our clinic with worsening axial back pain and neurogenic claudication. Twelve years prior to presentation at our clinic, the patient had an L4-S1 fusion for lumbar spondylosis. Following that procedure, he continued to have pain and had tried multiple epidural steroid injections and physical therapy with minimal relief. An L3/4 extreme lateral interbody fusion and posterior lumbar fusion extension to L3 for severe lumbar spinal stenosis secondary to adjacent segment disease at L3/L4 were performed. During this procedure, a durotomy occurred in a relatively inaccessible location below the axilla of the left L4 nerve root, requiring secondary repair with an on-lay DuraGen dural substitute, autologous fat graft, and fibrin dural sealant. The patient's symptoms improved after surgery. Five months later, the patient presented with severe pain radiating down his left buttocks into his calf, worse with standing and Valsalva maneuvers. The physical exam was notable for a positive straight leg test on the left. Electromyography (EMG) of the left lower extremity confirmed left L5 radiculopathy. Magnetic resonance imaging (MRI) of his lumbar spine demonstrated L4-L5 foraminal stenosis, an L3-L5 extradural fluid collection, and abnormal lumbar spinal canal enhancement. There was a lateral displacement of the nerve roots in the lower lumbar spinal canal with an "empty sac sign" (Figure 1). CT myelogram revealed stable tethering and clumping of the cauda equina nerve roots at L2-L3 concerning adhesions with no uptake into the L3-5 fluid collection (Figure 2).

**Figure 3:**
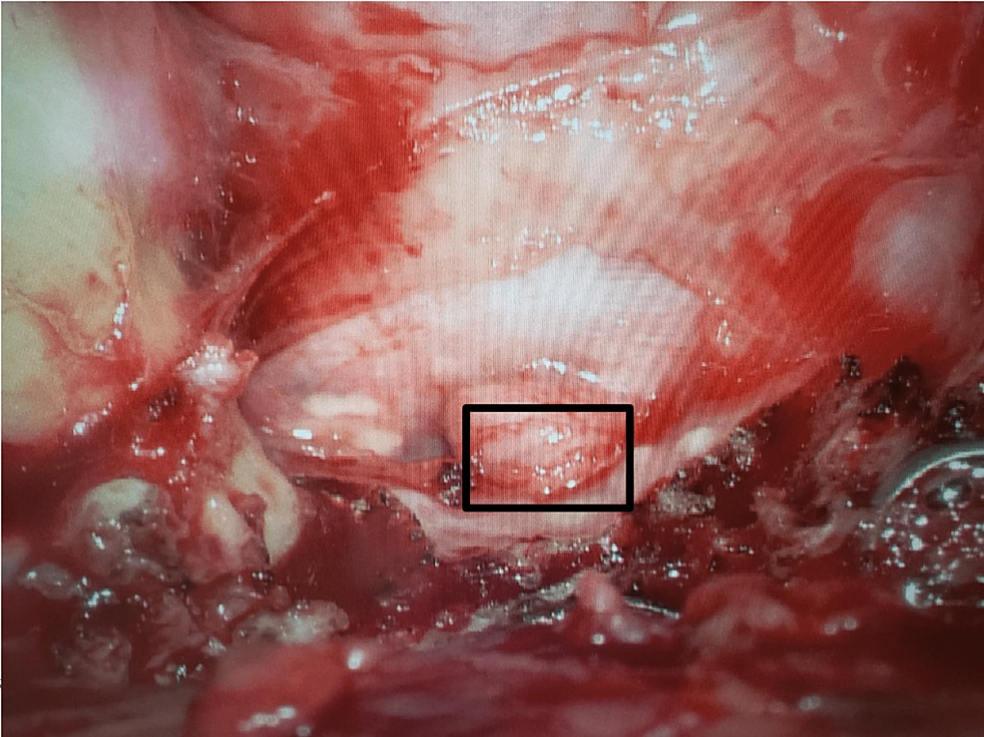
Intraoperative image that demonstrates herniation of the left L5 nerve root (box) through the prior durotomy defect.

**Figure 4:**
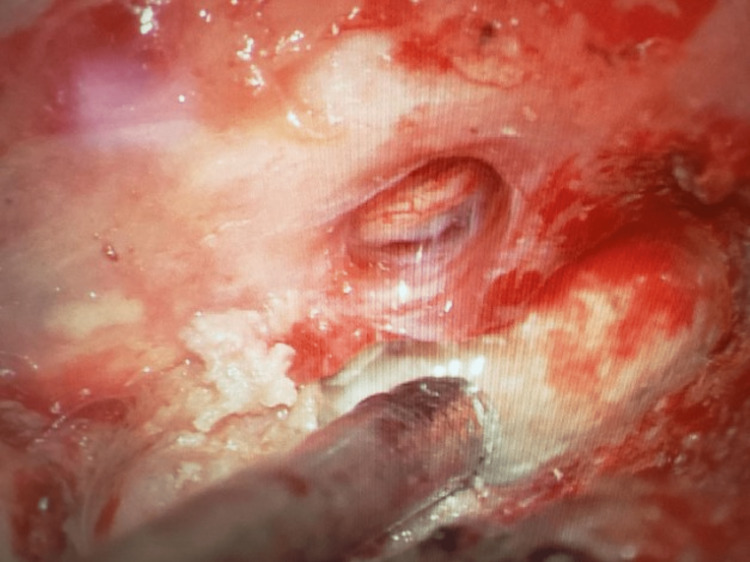
Intraoperative image after reduction of the left L5 nerve root through the defect.

**Figure 5:**
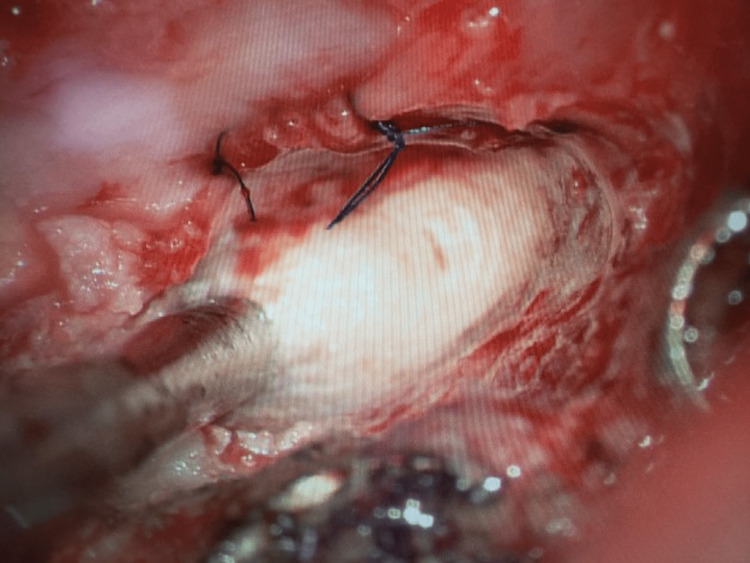
Final intraoperative image after primary repair of the durotomy defect and reduction of the nerve root.

While both imaging studies were read as consistent with arachnoiditis, the patient's worsening symptoms with coughing and standing were not. Exploratory surgery identified a herniated L5 nerve root through the prior durotomy defect (Figure 3). The left L5 nerve root was reduced, the durotomy closed primarily with a non-absorbable suture, and a lumbar drain was placed (Figure 4, 5). The patient's radicular pain improved immediately. The patient was discharged home on a postoperative day seven with no reported episodes of radicular pain since.

## Discussion

While incidental durotomies are a common complication of spinal surgery, postoperative neurological deficits are rare and feared complications, with a reported incidence of 0-2% [13]. Six months after surgery, our patient reported severe radicular pain worse with sneezing, coughing, and standing. The initial MRI lumbar revealed an empty sac sign, a focal thickening of the meninges such that little to no nerve roots are visible within the subarachnoid space [14]. An empty sac sign is characteristic of spinal arachnoiditis, a rare disease most common in patients who have undergone multiple lumbar surgeries or myelograms and manifesting as leg and back pain at various presentations [15-20].

The delayed presentation, history of spine surgery, and empty sac sign on imaging aligned with arachnoiditis. However, the worsening of our patient's radicular symptoms with standing and coughing was unusual and did not support an inflammatory cause. Likewise, the patient's history of prior spine surgeries, the most recent complicated by a durotomy defect with secondary closure, pointed to a potential structural cause. Identifying a herniated nerve root in exploratory surgery helped explain the patient's symptomatology. The increased pressure on the lumbar thecal sac with standing and coughing placed more pressure on the herniated nerve root, worsening the radicular pain. We suspect the radicular pain was delayed due to the gradual herniation of the L5 nerve root through the durotomy defect until it became entrapped. The empty sac sign on the initial MRI was due to the nerve roots being tethered to the periphery by the herniated nerve root. If arachnoiditis had been the primary cause, the patient would not have improved with surgical repair. Likewise, if only medical management had been used, the patient would likely have lived with severe chronic radiculopathy.

## Conclusions

This is a unique case of a patient with a history of epidural steroid injections and multiple spinal surgeries who presented with delayed radicular pain six months after an incidental durotomy was repaired with secondary closure. Initial imaging identified an empty sac sign suggestive of arachnoiditis, but the patient's worsening symptoms with standing and coughing pointed to an alternative cause. Exploratory surgery identified a herniated nerve root entrapped in the prior durotomy defect. This case highlights the importance of considering alternative diagnoses when imaging findings do not fit the clinical picture. Though incidental durotomies are common following spinal surgeries, this presentation of a herniated nerve root through a durotomy defect emphasizes the preference for and importance of primary closure in durotomy repair.
